# Two Novel *HSD17B4* Heterozygous Mutations in Association With D-Bifunctional Protein Deficiency: A Case Report and Literature Review

**DOI:** 10.3389/fped.2021.679597

**Published:** 2021-07-23

**Authors:** Si Chen, Linrun Du, Yihui Lei, Yuanyuan Lin, Shangqin Chen, Yanli Liu

**Affiliations:** Department of Neonatology, The Second Affiliated Hospital and Yuying Children's Hospital of Wenzhou Medical University, Wenzhou, China

**Keywords:** D-bifunctional protein deficiency, neonatal seizures, *HSD17B4*, peroxisomal disease, hypotonia

## Abstract

**Background:** D-Bifunctional protein deficiency (D-BPD) is an autosomal recessive disorder caused by peroxisomal β-oxidation defects. According to the different activities of 2-enoyl-CoA hydratase and 3-hydroxyacyl-CoA dehydrogenase protein units, D-bifunctional protein defects can be divided into four types. The typical symptoms include hypotonia and seizures. The gene that encodes D-BP was *HSD17B4*, which is located in chromosome 5q23.1.

**Case Presentation:** We report the first case of D-BPD in a Chinese patient with neonatal onset. Cosmetic malformations, severe hypotonia and seizures are prominent. The blood bile acid profile showed increased taurocholic acid, glycocholic acid, and taurochenodeoxycholic acid. Very-long-chain fatty acids (VLCFAs) revealed significant increases in hexacosanoic acid (C26:0), tetracosanoic acid/docosanoic acid (C24:0/C22:0), and hexacosanoic acid/docosanoic acid (C26:0/C22:0). Cranial MRI revealed bilateral hemispheric and callosal dysplasia, with schizencephaly in the right hemisphere. EEG showed loss of sleep–wake cycle and epileptiform discharge. Other examinations include abnormal brainstem auditory evoked potentials (BAEPs) and temporal pigmented spots on the optic disc in the right eye. After analysis by whole-exome sequencing, heterozygous c.972+1G>T in the paternal allele and c.727T>A (p.W243R) in the maternal allele were discovered. He was treated with respiratory support, formula nasogastric feeding, and antiepileptic therapy during hospitalization and died at home due to food refusal and respiratory failure at the age of 5 months.

**Conclusions:** Whole-exome sequencing should be performed in time to confirm the diagnosis when the newborn presents hypotonia, seizures, and associated cosmetic malformations. There is still a lack of effective radical treatment. Supportive care is the main treatment, aiming at controlling symptoms of central nervous system like seizures and improving nutrition and growth. The disease has a poor outcome, and infants often die of respiratory failure within 2 years of age. In addition, heterozygous deletion variant c.972+1G>T and missense mutations c.727T>A (p.W243R) are newly discovered pathogenic variants that deserve further study.

## Introduction

Peroxisomal diseases are divided into two categories: peroxisome biogenesis diseases and single peroxisomal enzyme/transporter defects. D-Bifunctional protein deficiency (D-BPD) (OMIM261515) is an autosomal recessive disorder caused by peroxisomal β-oxidation defects (1–3). The prevalence of peroxisomal defects has been roughly estimated at 1:30,000 and of D-BPD at 1:100,000 (4). The first D-BPD patient was reported in 1989 by Watkins et al. (5), and it was found that the true defect in this patient is the level of the D-BP but not the level of the L-BP in 1999 (6), since D-BP was discovered in 1996 (7). D-Bifunctional protein (D-BP) is a steroid metabolizing enzyme situated only in mammalian peroxisomes and is widely distributed in various organs throughout the body. D-BP contains three functional units: a 2-enoyl-CoA hydratase unit, a 3-hydroxyacyl-CoA dehydrogenase unit, and a sterol carrier protein 2 unit. The three functional units of D-BP are essential for the decomposition of very-long-chain fatty acids (VLCFAs), α-methyl branched-chain fatty acids, and bile acid intermediates such as dihydroxycholanic acid (DHCA) and trihydroxycholanic acid (THCA) (7–11). D-BP participates in peroxisomal β-oxidation reactions, specifically catalyzing the second (dehydration) and third (dehydrogenation) reactions of the peroxisomal β-oxidation of D-3-hydroxyacyl-CoA.

D-BPD has been classified into three types: type I, deficiency of 2-enoyl-CoA hydratase unit, and 3-hydroxyacyl-CoA dehydrogenase unit; type II, isolated hydratase deficiency; and type III, isolated dehydrogenase deficiency (2). The three profiles had similar clinical characteristics but different severities. The Kaplan–Meier survival analysis shows that type I deficient patients had the most severe symptoms, with 6.9 months as a mean age of death, while type II deficient patients and type III deficient patients had longer mean age of death, which was 10.7 and 17.6 months, respectively. And type I deficient patients would die within the first 14 months of life and had a poorer prognosis than patients with type II or III D-BPD (4). A type IV phenotype has been proposed based on the presence of missense mutations in each enzyme domain, and this mutation results in significantly reduced but detectable hydratase and dehydratase activities of D-BP, termed juvenile-type D-BPD (12). Absence of one or both of these enzymes (hydratase and dehydrogenase) invariably leads to impaired catabolism of VLCFA, DHCA, THCA, and pristanic acid. So accumulation of VLCFA, DHCA, and THCA is a prominent manifestation of D-BPD and can be confirmed by functional analysis and mutational analysis of enzyme activity in patient cells, usually skin fibroblasts (4).

D-BPD may develop in neonates, adolescents, or adults, but the onset of symptoms usually occurs in the neonatal period. Hypotonia (98%) and seizures (93%) usually occur during the first month of life, and patients usually die within 2 years after birth (4).

The gene that encodes D-BP was *HSD17B4* (13), which is located in chromosome 5q23.1 and was found to be more than 100 kbp in length. The gene consists of 24 exons and 23 introns. Homozygous or compound heterozygous mutations in *HSD17B4* gene cause D-BPD. In addition, *HSD17B4* is also one of the genes responsible for Perrault syndrome (PRLTS), manifesting with sensorineural hearing loss in both sexes, primary ovarian insufficiency in females, and neurological feature. Chen et al. (14) gave a report of a PRLTS family in China and found an *HSD17B4* mutation c298G>T (p.A100S) to confirm the relationship. Here, we report the first case of a Chinese neonatal-onset D-BPD patient with novel compound heterozygous mutations of *HSD17B4* (OMIM601860), including a splicing mutation and a missense mutation, detected by exome sequencing. And we have also summarized the clinical and genetic characteristics of the patient.

## Case Presentation

**Figure 1:**
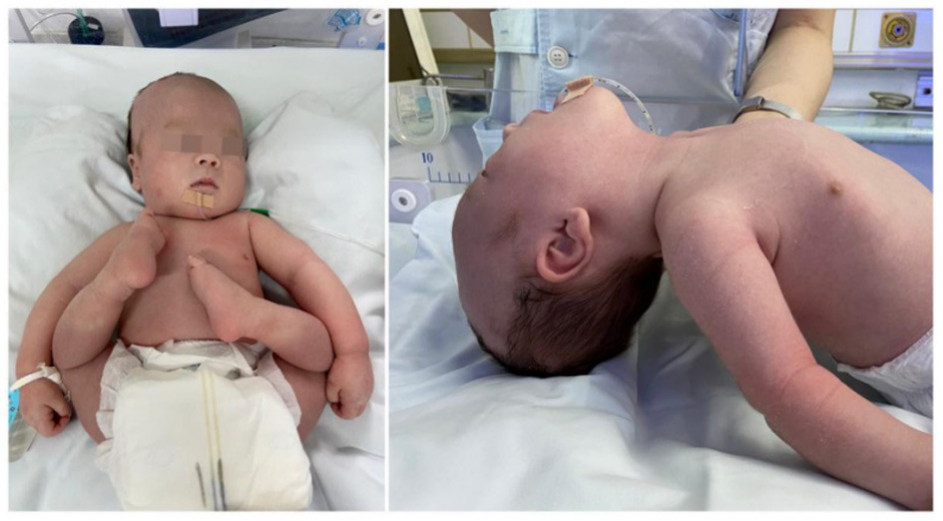
The patient with severe hypotonia and facial dysmorphism.

A 1-day-old male proband was hospitalized in the Department of Neonatology, Yuying Children's Hospital Affiliated to Wenzhou Medical University, in August 2020 due to “shortness of breath and hypotonia for 1 day, convulsions for 8 h.” The child was G3P2, born at the 39 weeks of gestation, singleton, by cesarean section due to “decreased fetal movement.” There were no placental, umbilical cord, or amniotic fluid abnormalities, and the Apgar scores were all 8 at 1, 5, and 10 min (−1 each for respiration and muscle tone). The patient's birth weight was 2,900 g (25th percentile), and length was 50 cm (25th−50th percentile). The patient has non-consanguineous parents, a healthy 11-year-old sister, and no history of familial genetic diseases. Convulsions and hypotonia (Figure 1) were found on the first day of life. Convulsions were characterized by fist clenching, eye gazing, and cyanosis of lips, which lasted for 10 s and resolved spontaneously. The infant was conscious during the interictal period but had poor responses including no spontaneous activity, no eyes pursuit, or normal sucking and swallowing. On examination, the infant was found to have craniofacial deformities, which showed a long head deformity (158 mm), high forehead, wide eye distance, and high arch of the palate, in addition to varus of both feet and left cryptorchidism. Basic reflexes (swallowing, sucking, and cough) were also depressed.

After admission, the patient was given oxygen inhalation with hood, formula nasogastric feeding, phenobarbital injection for stopping convulsion, intravenous fluid support, and other treatments. On the 1st day after admission, the patient had a convulsion, manifested as described before. The frequency of convulsive seizures gradually increased, and the infant's reaction did not improve. On the 9th day after birth, apnea was caused by sputum blockage, and the patient was given tracheal intubation and mechanical ventilation support. On the 11th day after birth, the patient developed fever with elevated C-reactive protein (CRP), considering sepsis, and was given anti-infection treatment. At 14 days after birth, the infant had frequent convulsive seizures, and levetiracetam oral solution was administered and gradually increased, but convulsions could not be controlled. At 21 days after birth, a midazolam injection was maintained for 1 week, and the dose was gradually increased, but the convulsion still could not be controlled. At 22 days after birth, levetiracetam tablets were increased to 60 mg/kg/day. On day 28, the endotracheal tube was withdrawn and changed to hood oxygen support again. After 35 days of life, he was treated with sodium valproate oral solution. Oxygen was withdrawn at 40 days after birth. Antiepileptic therapy was adjusted during hospitalization with the assistance of a pediatric neurologist. At 42 days after birth, the infant still had convulsive seizures more than 10 times a day under antiepileptic treatment with levetiracetam, topiramate tablets, and sodium valproate oral solution. The patient was discharged with drugs after the family learned nasogastric feeding. At the age of 5 months, the child died at home due to food refusal and respiratory failure.

### Laboratory Tests

Laboratory tests after birth showed no significant abnormalities in blood routine and CRP (transient increase due to infection), liver and kidney function, electrolytes, infectious disease screening, TORCH, coagulation function, blood glucose, blood ammonia, blood lactate, and blood gas analysis. The cerebrospinal fluid routine was normal with negative culture. Peripheral blood chromosome was 46XY. Blood lipids and cholesterol showed no abnormalities; blood tandem mass spectrometry showed no abnormalities in the measured amino acids and acylcarnitines. Urine tandem mass spectrometry reported increased methylcrotonylglycine. The blood bile acid profile showed that taurocholic acid 6.490 μmol/L (reference range ≤ 0.31 μmol/L), glycocholic acid 6.180 μmol/L (reference range ≤ 4.96 μmol/L), and taurochenodeoxycholic acid 2.09 μmol/L (reference range ≤ 0.8 μmol/L) were significantly increased, with the normal remaining bile acids and total bile acids. Peroxisome parameters, i.e., VLCFA, revealed significant increases in hexacosanoic acid (C26:0) 10.58 nmol/ml (normal range ≤ 1.30 nmol/ml), tetracosanoic acid/docosanoic acid (C24:0/C22:0) 2.07 (normal range ≤ 1.39), and hexacosanoic acid/docosanoic acid (C26:0/C22:0) 0.291 (normal range ≤ 0.023).

### Imaging and Electroencephalogram Examination

The first cranial magnetic resonance imaging (MRI) examination showed no abnormalities at 7 days after birth. The second cranial MRI revealed bilateral hemispheric and callosal dysplasia at 31 days after birth, with schizencephaly in the right hemisphere. On the 2nd day after birth, the background activity of amplitude-integrated electroencephalogram (aEEG) was continuous normal voltage, but no sleep–wake cycle was observed. Examination of aEEG on the 3rd day after birth showed recovered sleep–wake cycle, but subclinical convulsive seizures were observed three times. On postnatal day 7, clinical convulsive seizures were observed on aEEG, and a single seizure showed sharp slow wave firing on the right fronto-central region and spreading to peripheral brain regions for 1–2 min. EEG at 21 days after birth showed abnormal background, and occasionally isolated asynchronous sharp waves predominantly bilateral on the fronto-central region, with the seizure type of peak dysrhythmia. The onset of convulsive seizures was more frequent than before, 2.75 times/h, with a single seizure time of 30 s to 1 min. Examination of aEEG and EEG at 1 month after birth showed no significant improvement. Other examinations discovered abnormal brainstem auditory evoked potentials (BAEPs) and temporal pigmented spots on the optic disc in the right eye. Cardiac ultrasound showed no abnormality; abdominal ultrasound revealed mild separation of the collecting systems of both kidneys; no denervated potential was observed in the muscles examined by electromyography (EMG), and the number of muscle units (MUs) decreased during re-contraction, showing a small amount—simple phase or simple phase.

### Mutation Analysis of *HSD17B4* Gene

**Figure 2:**
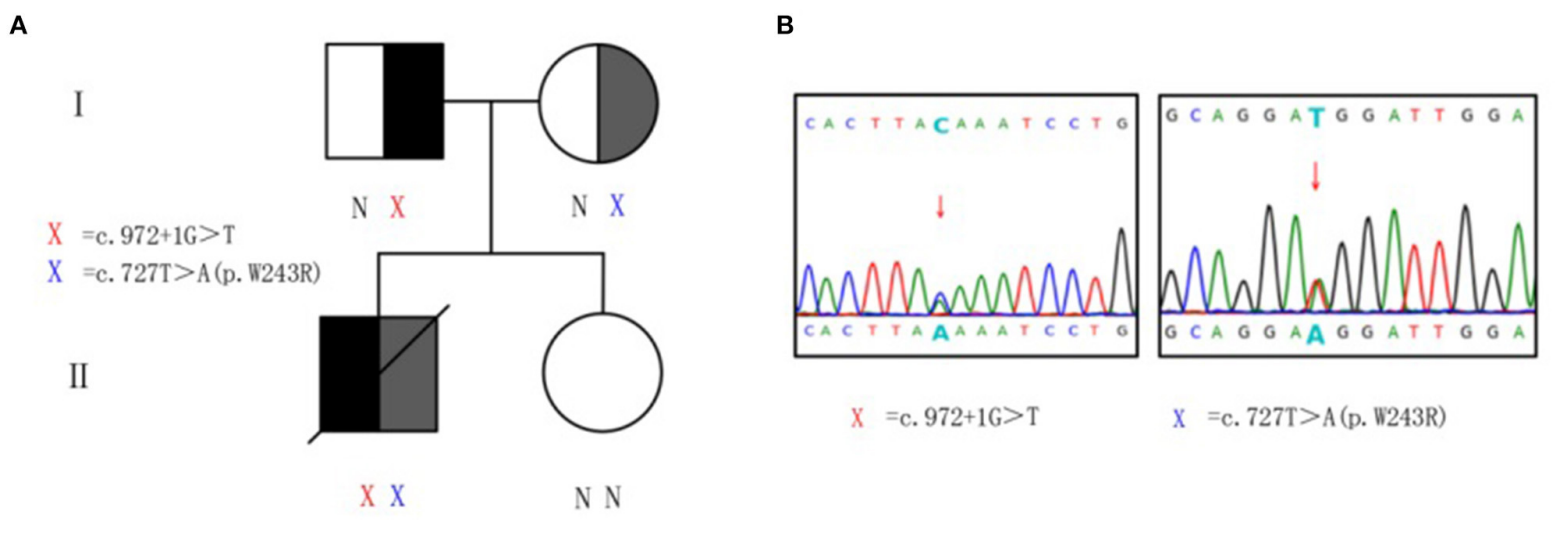
Heterozygous mutations of *HSD17B4* in the family. **(A)** Pedigree of family with D-bifunctional protein (DBP) deficiency. X, variant; N, normal. **(B)** Sanger sequencing validation of *HSD17B4* variants identified by exome sequencing. For the mutated gene c.972+1G>T, the sequencing validation is reversed, and the complementary chain of G>T is C>A. Genomic DNA was amplified for sequencing with primers flanking intron 12 and exon 10. Arrow indicates heterozygous mutations. Red X, c.972+1G>T at chr5:119496647–119496647. Blue X, c.727T>A (p.W243R) at chr5:119492112–119492112.

Genomic DNA samples were prepared from peripheral blood leukocytes of patients and subjected to whole-exome-generation sequencing analysis. Two heterozygous mutations were monitored in the subject on gene *HSD17B4* in 5q23.1. They are c.972+1G>T heterozygous mutation and c.727T>A heterozygous mutation (Figure 2). The splicing mutation c.972+1G>T is located in intron 12 and may lead to functional defects in hydratase unit; the non-synonymous single-nucleotide variant (SNV) mutation c.727T>A (p.W243R) is located in exon 10, resulting in changes in the short-chain dehydrogenase unit. The above two variants were not found in the Chinese population-specific database “Shenzhou Genome Database,” human exon database (ExAC), reference population 1,000 Genomes (1000G), and population genome mutation frequency database (gnomAD).

Peripheral venous blood was collected from the proband's parents and sister for validation and source analysis by whole-exome sequencing. The results suggested that his father was a carrier of the c.972+1G>T mutation and his mother was a carrier of the c.727T>A (p.W243R) mutation (Figure 2). Thus, the patient contained a mixed heterozygous mutation in *HSD17B4*, including c.972+1G>T in the paternal allele and c.727T>A (p.W243R) in the maternal allele. According to the 2015 American College of Medical Genetics and Genomics (ACMG) guidelines and the application recommendations of ClinGen Sequence Variant Interpretation (SVI) expert group for the guidelines (15–17), it is suggested that these two variants are pathogenic variants. Combined with VLCFA, bile acid profile, and cranial MRI results, this variant was considered as an extremely pathogenic variant.

## Discussion

In this case report, the patient showed typical clinical abnormalities including cosmetic deformities (long head deformity, high forehead, wide eye distance, high palatal arch, and talipes varus). It is consistent with the literature reports that most patients with early onset had craniofacial deformities (1, 18–20). The infant had severe neonatal hypotonia and convulsions, without primitive reflex elicited on the 1st day after birth, which was consistent with the literature reports that convulsive seizures occurred within a few days after birth, generally starting on the 2nd day after birth (1, 3, 18–21). After the patient's family signed the informed consent form for antiepileptic drugs, a combination of antiepileptic drugs, including levetiracetam tablets, topiramate tablets, and sodium valproate oral solution, was administered successively and at the maximum dose within the safety range, but the patient still had more frequent convulsive seizures of more than 10 times a day. It is consistent with most literatures reporting that the antiepileptic effect of this triple drugs still cannot control convulsive seizures (20, 22). In addition, McMillan et al. (12) and Khan et al. (22) reported the presence of retinitis pigmentosa in patients with D-BPD. We performed two fundus examinations on the patient, both of which revealed temporal pigment spots on the optic disc in the right eye, and may progress to retinitis pigmentosa, which needs further follow-up. This may be because long-chain polyunsaturated fatty acids are important substrates for DHA biosynthesis, and their β-oxidation requires D-BP involvement. Therefore, the lack of D-BP will indirectly lead to DHA deficiency, ultimately affecting brain and retinal development. Bae et al. (18) reported that although DHA supplementation in patients could increase DHA levels, it still could not improve clinical outcomes in patients with DHA deficiency. And patients showed progressive visual impairment and brain deterioration despite early DHA supplementation (within a month) (18). In addition, the disease often causes abnormal BAEP and even leads to deafness. Children with infantile onset have a higher mortality rate according to the above case presentations in the literature. In our case, we performed BAEP for two times in the patient, both of which suggested that the patient had binaural hearing impairment.

It has been reported in the literature that cranial MRI in adulthood revealed cerebellar atrophy and ataxia (23), while neonatal cranial MRI revealed no significant brain atrophy (24). Our patient's cranial MRI showed shallow sulci, local widening and deepening of the lateral fissure cistern of the right cerebral hemisphere, extensive hyperintense white matter changes in the cerebral hemisphere on T2WI, and dysplasia of the corpus callosum, which were similar to the clinical report that MRI in children with D-BDP showed different severities of lateral fissure, peripheral multiple microgyria, and delayed myelination (3, 18, 21).

The proband in this study was examined for plasma bile acid profile at 32 days after birth; and it was found that taurocholic acid, glycocholic acid, and taurochenodeoxycholic acid levels were significantly increased, which was consistent with the manifestations of bile acid metabolism disorders in D-BPD as reported in the literature (25).

The biochemical diagnosis of D-BPD is based on the accumulation of VLCFA, DHCA, THCA, and pristanic acid in plasma. Biochemical analysis requires the supplementation of erythrocyte acetal phospholipids, phytanic acid, and bile acid intermediates in plasma in order to make a preliminary distinction for possible diagnosis (19). There was a good correlation between patient survival and the level of C26:0 in fibroblasts. Patients who survived had more residual enzyme activity and lower 26:0 levels. In patients surviving more than 4 years, no abnormal plasma fibrinogen was found (3). In recent years, it has become increasingly clear that, despite the presence of peroxisomal disease, there are conditions in which very-low-density lipoprotein cholesterol and/or other peroxisomal metabolites are normal. Landau et al. (21) reported that the levels of VLCFA (including phytic acid) were within the normal range in two of three patients. It is a pity that we did not test the patient's C26:0 level of fibroblasts, but examination of serum VLCFA levels 33 days after birth suggested that the C26:0, C26:0/C22:0, and C24:0/C22:0 ratios were higher than normal levels, which was consistent with those reported in the literature (18, 19, 26). However, peroxisome metabolism is often at normal levels in the neonatal period, and common hematuria metabolic screening did not detect VLCFA metabolism abnormalities, so the diagnosis of similar cases will be easily missed. In that case, whole-exome sequencing is recommended for suspicious cases. A report of Lines et al. (23) states that all reported diagnoses of D-BPD in adolescents are done by whole-exome sequencing rather than by traditional clinical means.

To date, all D-BPD patients reported in the literature are homozygous or compound heterozygous for *HSD17B4* mutations. Our patient had a heterozygous deletion variant c.972+1G>T and missense mutations c.727T>A (p.W243R). These two mutations have never been reported before.

D-BPD type I defects are often associated with nonsense mutations, frameshift mutations, or in-frame deletions of 20 or more residues in the dehydrogenase domain; and in patients with type I deficiency, *HSD17B4* protein is almost always undetectable in fibroblasts. Defects are associated with missense mutations or in-frame deletions in the hydratase domain, and type III defects are associated with missense mutations or single amino acid deletions in the dehydrogenase domain (27, 28). The expression of mutant *HSD17B4* protein was severely reduced in compound heterozygotes. D-BPD types includes type I, type II, type III, and type IV, among which type III is the most common D-BPD type in infantile onset (<2 years) patients. In our case, the exon mutation c.727T>A (p.W243R) of *HSD17B4* leads to a disturbance in SDR domain of dehydrogenase unit, and intron mutation c.972+1G>T may lead to splicing abnormal in hydratase unit. Regarding intron mutation pathogenesis, it has been shown that fetus with homozygous mutations in intron IVS5+1G>C of *HSD17B4* has increased VLCFA levels (29). In our case, although we did not detect the activity of the two units of D-BP, we suggest the patient may have had type I D-BPD.

We report the first Chinese patient with bifunctional protein deficiency, analysis of this variant according to the ACMG guidelines suggests an extremely pathogenic variant, and the child's clinical presentation is compatible with D-BPD. The mutations found in our case have not been previously reported worldwide. The mutation type was compound heterozygous and may result in D-BPD type I, although we did not measure *HSD17B4* protein activity and could not identify the specific D-BP type, which is a limitation of this report. Given the early onset and severe disease of this patient, it is estimated that there is a high possibility of almost complete lack of *HSD17B4* protein activity. In terms of treatment, the disease is based on symptomatic and supportive treatment. There is still a lack of effective radical treatment, and children often die of respiratory failure within 2 years of age.

## Conclusions

Our case provides clinical features of a rare D-BPD and a new type of *HSD17B4* gene mutation to provide a reference for early diagnosis. The presence of hypotonia and intractable epilepsy in infancy, associated with cosmetic deformities, especially if cranial MRI is associated with polymicrogyria, severe developmental delay, hearing loss, or primary adrenal insufficiency, regardless of their VLCFA condition, should be considered for this disease. The disease has a poor outcome, and infants often die of respiratory failure within 2 years of age. In addition, heterozygous deletion variant c.972+1G>T and missense mutation c.727T>A (p.W243R) are newly discovered pathogenic variants that deserve further study.

## Data Availability Statement

The original contributions presented in the study are included in the article/Supplementary Material, further inquiries can be directed to the corresponding author/s.

## Ethics Statement

The studies involving human participants were reviewed and approved by 2021-K-12-01. Written informed consent to participate in this study was provided by the participants' legal guardian/next of kin. Written informed consent was obtained from the minor(s)' legal guardian/next of kin for the publication of any potentially identifiable images or data included in this article.

## Author Contributions

SiC, LD, YiL, YuL, ShC, and YaL drafted the manuscript or revised it critically for important intellectual content, provided the final approval of the version to be published, and agreed to be accountable for all aspects of the work in ensuring that questions related to the accuracy or integrity of any part of the work were appropriately investigated and resolved. All authors contributed to the article and approved the submitted version.

## Conflict of Interest

The authors declare that the research was conducted in the absence of any commercial or financial relationships that could be construed as a potential conflict of interest.

## Publisher's Note

All claims expressed in this article are solely those of the authors and do not necessarily represent those of their affiliated organizations, or those of the publisher, the editors and the reviewers. Any product that may be evaluated in this article, or claim that may be made by its manufacturer, is not guaranteed or endorsed by the publisher.
