# Supplementary material for: Two Novel HSD17B4 Heterozygous Mutations in Association With D-Bifunctional Protein Deficiency: A Case Report and Literature Review
Source: Front Pediatr. 2021 Jul 23;9:679597. doi: 10.3389/fped.2021.679597 (PMC8342883; doi:10.3389/fped.2021.679597)
Supplement: Supplementary file 1 [file Table_1.DOCX]

**The CARE guidelines checklist**

1. **Title** – Two novel HSD17B4 heterozygous mutations in association with D-bifunctional protein deficiency-a case report and literature review
2. **Key Words** – D-bifunctional protein deficiency； neonatal seizures； HSD17B4
3. **Abstract**
4. **Introduction** – D-bifunctional protein deficiency (D-BPD) is an autosomal recessive disorder caused by peroxisomal β-oxidation defects. According to the different activity of 2-enoyl-CoA hydratase and 3-hydroxyacyl-CoA dehydrogenase protein units, D-bifunctional protein defects can be divided into four types. The typical symptoms includes hypotonia and seizures. The gene that encodes D-BP was HSD17B4, which is located on chromosome 5q23.1.
5. **Case Presentation:**

- **The main symptoms of the patient**: cosmetic malformations, early onset of hypotonia, poor responses and frequent seizures
- **The main clinical findings**: The blood bile acid profile showed increased taurocholic acid, glycocholic acid and taurochenodeoxycholic acid. VLCFA revealed significant increases in docosanoic acid, docosanoic acid/docosanoic acid, and docosanoic acid/docosanoic acid. Cranial MRI revealed bilateral hemispheric and callosal dysplasia at 31 days after birth, with schizencephaly in the right hemisphere. EEG showed loss of sleep-wake cycle and epileptiform discharge. Other examinations discovered abnormal brainstem auditory evoked potentials(BAEP) and temporal pigmented spots on the optic disc in the right eye. the patient contained a mixed heterozygous mutation in HSD17B4, including c.972 + 1G > T in the paternal allele and c.727T > A (p.W243R) in the maternal allele.
- **The main diagnoses and interventions**: The patient was diagnosed D-bifunctional protein deficiency. He was treated with respiratory support, formula nasogastric feeding, and antiepileptic therapy.
- **The main outcomes**: The patient was discharged with drugs after the family learned nasogastric feeding. At the age of 5 months, the child died at home due to food refusal and respiratory failure.

1. **conclusions**- D-bifunctional protein deficiency is a rare disease, and whole-exome sequencing should be performed in time to confirm the diagnosis when the newborn presents hypotonia, seizures, and associated cosmetic malformations. There is still a lack of effective radical treatment. Supportive care is the main treatment, aiming at controlling symptoms of central nervous system like seizures, and improving nutrition and growth. The children often die of respiratory failure within 2 years of age.
2. **Introduction** D-bifunctional protein deficiency (D-BPD) (OMIM261515) is an autosomal recessive disorder caused by peroxisomal β-oxidation defects1-3. The prevalence of peroxisomal defects have been roughly estimated at 1:30,000 and of DBPD at 1:100,0004. The first D-BPD patient was reported in 1989 by Watkins5, and was found that the true defect in this patient is the level of the D-BP but not the level of the L-BP in 1999 6, since D-BP was discovered in 19967. D-bifunctional protein (D-BP) is a steroid metabolizing enzyme situated only in mammalian peroxisomes and is widely distributed in various organs throughout the body. D-BP contains three functional units, a 2-enoyl-CoA hydratase unit, a 3-hydroxyacyl-CoA dehydrogenase unit, and a sterol carrier protein 2 unit. The three functional units of D-BP are essential for the decomposition of very long-chain fatty acids (VLCFA), α-methyl branched-chain fatty acids, and bile acid intermediates such as dihydroxycholanic acid (DHCA) and trihydroxycholanic acid (THCA)7,8-11. D-BP participates in peroxisomal β-oxidation reactions, specifically catalyzing the second (dehydration) and third (dehydrogenation) reactions of the peroxisomal beta-oxidation of D-3 hydroxyacyl-CoA. D-BPD has been classified into three types: type I, deficiency of 2-enoyl-CoA hydratase unit and 3-hydroxyacyl-CoA dehydrogenase unit; type II,isolated hydratase deficiency; and type III, isolated dehydrogenase deficiency12。The three profiles had similar clinical characteristics but different severities. The Kaplan-Meier survival analysis shows that type I-deficient patients had the most severe symptoms, with 6.9 months as a mean age of death; while type II-deficient patients and type III-deficient patients had longer mean age of death, which was 10.7 and 17.6 months respectively. And type I-deficient patients would die within the first 14 months of life and had a poorer prognosis compared with patients with type II or III DBPD4. A type IV phenotype has been proposed based on the presence of missense mutations in each enzyme domain, and this mutation results in significantly reduced but detectable hydratase and dehydratase activities of DBP, termed juvenile-type D-BPD12. Absence of one or both of these enzymes (hydratase and dehydrogenase) invariably leads to impaired catabolism of VLCFA, DHCA, THCA, and ortho-amino acids. So accumulation of VLCFA, DHCA, and THCA is a prominent manifestation of D-BPD and can be confirmed by functional analysis and mutational analysis of enzyme activity in patient cells, usually skin fibroblasts4. D-BPD may develop in neonates, adolescents, or adults, but the onset of symptoms usually occurs in the neonatal period. Hypotonia (98%) and seizures (93%) usually occur during the first month of life, and patients usually die within 2 years after birth4. The gene that encodes D-BP was HSD17B413, which is located on chromosome 5q23.1 and was found to be more than 100 kbp in length. The gene consists of 24 exons and 23 introns. Homozygous or compound heterozygous mutations in the HSD17B4 gene cause D-BPD. Here, we report the first case of a Chinese neonatal-onset D-BPD patient with novel compound heterozygous mutations of HSD17B4 (OMIM601860), including a splice mutation and a missense mutation, detected by exome sequencing. And we have also summarized the clinical and genetic characteristics of the patient.
3. **Patient Information**
4. **Demographic information**: The patient was a one-day-old Han Chinese male infant.
5. **Main symptoms of the patient**: cosmetic malformations, early onset of hypotonia, poor responses and frequent convulsive seizures
6. **Medical, family, and psychosocial history**: The patient was born at 39th weeks of gestation via cesarean section. He was the second-born child of a healthy, non-consanguineous Chinese couple, with a a healthy 11-year-old sister. The birth weight was 2900gm. There were no placental, umbilical cord, or amniotic fluid abnormalities, and the Apgar scores were all 8 at 1 minute, 5 minutes, and 10 minutes (-1 each for respiration and muscle tone). The parents denied similar disease and inherited metabolic diseases among family members.
7. **Clinical Findings**: Convulsions and hypotonia were found on the first day of life. Convulsions were characterized by fist clenching, eye gazing, and cyanosis of lips, which lasted for tens of seconds and resolved spontaneously. The infant was conscious during the interictal period, but had poor responses including no spontaneous activity, no eyes pursuiting or normal sucking and swallowing. On examination, the infant was found to have craniofacial deformities, which showed a long head deformity (158mm), high forehead, wide eye distance, and high arch of the palate, in addition to varus of both feet and left cryptorchidism. Basic reflexes (swallowing, sucking and cough) were also depressed.
8. **Timeline**

| Time | Episodes |
| --- | --- |
| Day 1 after admission | oxygen inhalation, formula nasogastric feeding, intravenous fluid support and phenobarbital injection.  The patient had a convulsion, the frequency of convulsive seizures gradually increased, while the infant’s reaction was not improved. |
| Day 9 | Apnea was caused by sputum blockage, and the patient was given tracheal intubation and mechanical ventilation support. |
| Day 11 | The patient developed fever with elevated C-reactive protein (CRP), considering sepsis, and was given anti-infection treatment. |
| Day 14 | The infant had frequent convulsive seizures. Levetiracetam oral solution was administered and gradually increased, but convulsions could not be controlled. |
| Day 21 | Amidazolam injection was maintained for 1 week, and the dose was gradually increased, with levetiracetam tablets increased to 60mg/kg/d the second day, but the convulsion still could not be controlled. |
| Day 28 | The endotracheal tube was withdrawn and changed to hood oxygen support again. |
| Day 35 | He was treated with sodium valproate oral solution. |
| Day 40 | Oxygen was withdrew. |
| Day 42 | The infant still had convulsive seizures more than 10 times a day under antiepileptic treatment with levetiracetam, topiramate tablets, and sodium valproate oral solution. The patient was discharged with drugs after the family learned nasogastric feeding. |
| 5 months old | the child died at home. |

1. **Diagnostic Assessment**
2. Diagnostic methods: PE; laboratory testing including the blood bile acid profile, very long-chain fatty acids (VLCFA); amplitude integrated electroencephalogram(aEEG); brainstem auditory evoked potentials(BAEP) and fundus screening, magnetic resonance imaging(MRI), tandem mass spectrum, gene sequencing
3. Diagnostic challenges: No
4. Diagnostic reasoning: clinical presentation, VLCFA level, BAEP and fundus screening, MRI, gene sequencing
5. Prognostic characteristics: poor prognosis
6. **Therapeutic Intervention**
7. Types of intervention

-Administration of intervention: Supportive care and antiepileptic treatment

-Changes in intervention: The infant still had uncontrollable convulsive seizures, with hypotonia and poor reaction

1. **Follow-up and Outcomes**
2. Summarize the clinical course of all follow-up visits including

-Clinician and patient-assessed outcomes: died at home at the age of 5 months.

-Important follow-up test results: No.

-Intervention adherence and tolerability: good

-Adverse and unanticipated events: no

**11. Discussion**

1. The strengths and limitations of the management of this case: The case has been closely observed by author through patient care, with enough experimental data and imaging data to support the diagnosis. No limitations.
2. The relevant medical literature: Several similar cases have been reported worldwide.
3. The rationale for conclusions: Causal relationship
4. The main “take-away” lessons of this case report: Our case provides clinical features of a rare D-BPD and a new type of HSD17B4 gene mutation to provide a reference for early diagnosis. The presence of hypotonia and intractable epilepsy in infancy, and associated with cosmetic deformities, especially if cranial MRI is associated with polymicrogyria, severe developmental delay, with hearing loss, or primary adrenal insufficiency, regardless of their VLCFA condition, should be considered for this disease. The disease has a poor outcoming, and infants often die of respiratory failure within 2 years of age. In Addition, heterozygous deletion variant c. 972 + 1G > T and missense mutations c. 727T > A (p.W243R) are newly discovered pathogenic variants, which deserves further study.

**12. Patient Perspective:** Agree.

**13. Informed Consent:** Yes.
